# DLD is a potential therapeutic target for COVID-19 infection in diffuse large B-cell lymphoma patients

**DOI:** 10.1007/s10495-024-01959-0

**Published:** 2024-04-06

**Authors:** Can Chen, Dandan Kang, Zhenzhen Chen, Pengfei Shi, Yun Li, Shenxian Qian

**Affiliations:** 1 Department of Hematology, Affiliated Hangzhou First People’s Hospital, School of Medicine, Westlake University, Hangzhou, China; 2 School of Medicine, Zhejiang University, Hangzhou, China; 3 Team of neonatal & infant development, health and nutrition, NDHN. School of Biology and Pharmaceutical Engineering, Wuhan Polytechnic University, Wuhan, China; 4 Kindstar Global Precision Medicine Institute, Wuhan, China

**Keywords:** DLBCL, DLD, KDM1A, CD8 + T cells, Biomarker

## Abstract

Since the discovery of copper induces cell death(cuprotosis) in 2022, it has been one of the biggest research hotspots. cuprotosis related genes (CRGs) has been demonstrated to be a potential therapeutic target for cancer, however, the molecular mechanism of CRGs in coronavirus disease 2019 (COVID-19) infected in DLBCL patients has not been reported yet. Therefore, our research objective is first to elucidate the mechanism and role of CRGs in COVID-19. Secondly, we conducted univariate and multivariate analysis and machine learning to screen for CRGs with common expression differences in COVID-19 and DLBCL. Finally, the functional role and immune mechanism of genes in DLBCL were confirmed through cell experiments and immune analysis. The research results show that CRGs play an important role in the occurrence and development of COVID-19. Univariate analysis and machine learning confirm that dihydrolipoamide dehydrogenase (DLD) is the common key gene of COVID-19 and DLBCL. Inhibiting the expression of DLD can significantly inhibit the cycle progression and promote cell apoptosis of DLBCL cells and can target positive regulation of Lysine-specific demethylase 1 (LSD1, also known as KDM1A) to inhibit the proliferation of DLBCL cells and promote cell apoptosis. The immune analysis results show that high-expression of DLD may reduce T cell-mediated anti-tumor immunity by regulating immune infiltration of CD8 + T cells and positively regulating immune checkpoints LAG3 and CD276. Reducing the expression of DLD can effectively enhance T cell-mediated anti-tumor immunity, thereby clearing cancer cells and preventing cancer growth. In conclusion, DLD may be a potential therapeutic target for COVID-19 infection in DLBCL patients. Our research provides a theoretical basis for improving the clinical treatment of COVID-19 infection in DLBCL.

**Supplementary Information:**

The online version contains supplementary material available at 10.1007/s10495-024-01959-0.

## Introduce

According to a report, novel coronavirus can cause or aggravate cancer diseases with various clinical manifestations [1]. Coronavirus disease 2019 (COVID-19) is an acute respiratory infectious disease that is caused by SARS-CoV-2, which has spread rapidly worldwide [2]. SARS-CoV-2 is a single-stranded RNA β-coronavirus with a lipidic envelope, and four types of proteins that are involved in its pathogenicity, the remaining 20% of individuals progress to more severe conditions, although approximately 80% of SARS-CoV-2 infected individuals have a mild course of illness [3]. COVID-19 has caused millions of deaths worldwide, besides, some COVID-19 patients experience serious complications, including multiple organ failure, shock and systemic inflammatory response syndrome, and acute respiratory disease syndrome [4]. These manifestations are common in several high-risk populations, including obesity, elderly patients, pulmonary and cardiovascular diseases, hypertensive patients, cancer, and autoimmune diseases [5].

At present, a lot of studies indicate that some susceptibility factors that are caused by SARS-CoV-2 virus may promote the development and occurrence of Diffuse large B cell lymphoma (DLBC/DLBCL) [6]. DLBCL is the most common hematological malignancy in the world, and its incidence rate has risen rapidly in the last 20 years [7]. Compared to other types of cancer patients, DLBCL that is infected with SARS-CoV-2 exhibits higher mortality rates and more severe symptoms. This is attributed to the significantly reduced immune function of the two diseases that are mentioned above. DLBCL has also been reported as a high-risk factor for COVID-19 [8]. It has also been reported that patients with DLBCL have poor prognosis due to COVID-19 infection [9]. The common pathogenesis of COVID-19 that is caused by SARS-Cov-2 virus and DLBCL, however, is still unclear.

On March 17, 2022, a study that is published in the international academic journal Science that is showed that copper induces cell death (cuprotosis) is a novel cell death mechanism differing from known cell death mechanisms, it occurs via copper’s direct binding to lipoylation components of the tricarboxylic acid (TCA) cycle. This results in lipoylated protein aggregation and subsequent iron-sulfur cluster protein loss leading to proteotoxic stress and ultimately cell death [10]. Cuprotosis has been reported to be associated with various diseases, cuprotosis related genes (CRGs) has been demonstrated to be a potential therapeutic target for cancer, [11]. Currently, only one literature has reported the relationship between DLBCL and cuprotosis, the study elucidated the changes in CRGs at the transcriptome level of DLBCL, however, the specific mechanisms have not been elucidated [12]. During the disease’s initial stages, COVID-19 patients had increased serum levels of copper ions, which were associated with the inflammatory response mainly [13]. It was found that whole blood copper levels were significantly higher in COVID-19 patients with severe condition compared to those with non-severe condition [14]. Research has found that serum copper in COVID-19 patients can help predict patient prognosis, and supplementing copper adjuvants in patients diagnosed with copper deficiency may have a positive impact on disease outcomes [15]. Copper ion levels’ imbalance can disrupt certain mitochondrial metabolic enzymes, which are more toxic in cells with active respiration. These enzymes can induce cell death, namely cuprotosis [16]. However, the specific mechanism of cuprotosis and CRGs in COVID-19 has not been reported.

In summary, DLBCL is a risk factor for COVID-19, Compared to healthcare providers with COVID-19, DLBCL subjects with COVID-19 have more severe conditions and significantly higher risk of death [17]. CRGs has been demonstrated to be a potential therapeutic target for cancer [18], but its role in COVID-19 stays unclear. CRGs have been reported at the DLBCL transcription level, but the specific mechanism is not clear, and no relevant cell function experiments have been conducted to verify it [12]. Our study identified potential therapeutic targets that were related to cuprotosis in COVID-19 infected DLBCL patients and elucidated their immune and pathological mechanisms in DLBCL through cell experiments and bioinformatics analysis and provides the treatment and clinical diagnosis of DLBLC that has not been improved a basis.

## Materials and methods

### Microarray data acquisition and processing

The gene expression microarray dataset for DLD expression level and diagnostic performance evaluation includes GSE177477, GSE56315, GSE25638, GSE83632, TCGA-DLBC. The Cancer Genome Atlas (TCGA) database (https://www.cancer.gov/ccg/research/genome-sequencing/tcga) and The GEO dataset can be downloaded from the Gene Expression Comprehensive Database (GEO) (https://www.ncbi.nlm.nih.gov/geo/). The GSE177477 was obtained from GPL23195 platform, including 29 SARS CoV-2 positive case samples and 18 healthy control samples in respiratory tract samples of COVID-19 patients [19]. The GSE56315 dataset was obtained from the GPL570 platform, including 33 matched normal samples and 55 DLBCL samples. The GSE25638 dataset was obtained from the GPL570 platform, including 13 matched normal samples and 26 DLBCL samples. The GSE83632 dataset was obtained from the GPL5175 platform, including peripheral blood samples from 87 healthy donors and 76 DLBCL patients. TCGA-DLBC dataset includes 33 DLBCL samples and the Genotype-Tissue Expression (GTEx) dataset (https://www.gtexportal.org) includes 337 normal samples. Single cell data from GSE175510 [20]. We used the R 4.2.1 tool to evaluate prognostic risk assessment and gene expression levels by analyzing raw data from microarrays [21].

## GEO database and machine learning used to screen key CRGs of COVID-19

we obtained data from GEO177477 and performed gene expression analysis. After data standardization, If the data has no batch effect, it can be used as a batch of data for subsequent analysis, and use the Fold change(FC) and corrected *p* value to draw a volcanic map. The red dot in the figure represents the genes with significant difference up-regulated, and the blue dot represents the genes with significant difference down-regulated; Differential gene expression heat map, in which different colors represent the expression trend in different tissues. *P* < 0.05 and FC > 1.50 or < 0.67 were considered to have significant differences in genes [22]. A machine that learnt (Random Forest, RF) model was subsequently used to further screen and select the gene with the highest contribution to classification (IncNodePurity > 0.4) as the key gene [23].

### Function and pathway enrichment analysis

Metascape (https://metascape.org/gp/index.html#/main/step1) is a website for analyzing protein lists or gene, which is used to analyze the functional clustering of gene sets. R package ClusterProfiler package is used to analyze the gene set of Kyoto Encyclopedia of Genes (KEGG) and gene ontology (GO), *P* < 0.05 is considered significant [24]. Gene Set Enrichment Analysis (GSEA) was used to study the biological signaling pathways between low and high expression of Hub genes [25].

### DLD is a potential biomarker for COVID-19、DLBCL tissue、peripheral blood and scRNA-seq

We first used COVID-19 samples from the GSE177477 dataset and DLBCL samples from the GSE56315 dataset to analyze the key upregulated genes with significant differences between the two diseases. Then it was confirmed through TCGA and GSE25638 [26]. Due to the heterogeneity of clinical samples, we evaluated the expression of dihydrolipoamide dehydrogenase (DLD) in peripheral blood of DLBCL using the GSE83632 dataset. In order to further evaluate the expression of DLD at the level of DLBCL single cell transcriptome, we evaluated the expression level of DLD at the level of DLBCL single cell transcription through single cell data GSE175510 [20].

### The mutation landscape of DLD in DLBCL

The mutation of DLD in DLBCL was studied using the cBioPortal database. And analyzed the correlation between mutations and clinical information of DLBCL [27].

### Pathway enrichment analysis at the transcriptional level of DLD and mechanism analysis at the protein level

Firstly, GSEA was used to investigate the biological pathways between low-DLD expression and high-DLD [25]. Secondly, we use GeneMANIA (https://genemania.org/) that a PPI network centered on DLD was constructed, which included the correlation data of genetic interactions and protein, pathways, co localization, co expression and protein domain similarity. Then, GO function enrichment analysis were carried out for the gene centered on DLD constructed by GeneMANIA [28].

### Correlation analysis between DLD and immune cell infiltration, immune checkpoints and immune characteristic genes

The TIMER database is a comprehensive resource that can be used to evaluate the immune efficacy of various types of cancer systematically. We used TIMER to analyze the correlation between a large number of tumor and DLD infiltrating immune cells (CD4 + T cells, B cells, CD8 + T cells, neutrophils, dendritic cells and macrophages) in DLBCL [29]. The correlation between immune characteristic genes and immune checkpoints in DLBCL and DLD was studied through Spearman correlation analysis. A value with *p* < 0.05 is considered statistically to be significant, and the correlation coefficient’s absolute value is close to 1, indicating a stronger correlation [30].

### Functional analysis of KDM1A in DLBCL and correlation analysis between DLD and KDM1A

Lysine-specific demethylase 1 (LSD1, also known as KDM1A) has been found to be highly expressed in neurocytoma, colon cancer, breast cancer and other tumor types [31], and high expression is associated with poor tumor prognosis. Inhibiting the expression of KDM1A has been reported as a feasible tumor treatment strategy [32]. There have been studies reporting KDM1A’s high expression in human DLBCL tissue, but the specific mechanism has not been elucidated [33]. We first used the TCGA-DLBC dataset to confirm KDM1A’s significant upregulation in DLBCL. Then, we used TCGA-DLBC data to analyze the differential genes (*P* < 0.01 and Log2 > 4) that were up-regulated in DLBCL ranking, and used R soft 4.2.1 to analyze the GSE56315 to verify TCGA-DLBC dataset’s results. Utilize STRING database (https://string-db.org/) to obtain differential genes’ network diagram in DLBCL, and obtain key functional gene modules through the MCODE add-in in the Cytoscape software. Spearman correlation was used to analyze the correlation between DLBCL key MCODE genes and KDM1A. Metascape is used to analyze the pathways that are enriched by genes significantly correlated with KDM1A. Finally, we analyzed the correlation between DLD expression and KDM1A expression that was based on the TCGA-DLBC dataset [34].

### Construction of cell model for DLD interference

OCI-LY1 cells are sourced from the cell bank of Hangzhou First Hospital in Zhejiang Province. OCI-LY1 cells are cell lines of ABC type DLBCL. SiRNA is a chemically synthesized small molecule that serves as an important intermediate for gene silencing and sequence specific RNA degradation. It has special structural features such as a 3’ end hydroxyl group and a 5 ‘end phosphate group, with two free bases at the 3 ‘end of each of its two chains. It degrades mRNA through specific complementary binding with the target mRNA. We constructed two siRNAs (OCI-LY1 + DLD-siRNA1 and OCI-LY1 + DLD-siRNA2) based on the DLD sequence. After transfection into OCI-LY1 cells, the interference effect of these two siRNAs on DLD was verified through qPCR. A random sequence was used as the control group (OCI-LY1 + NC-siRNA), and the cell model of DLD interference was constructed by selecting one of the two siRNAs with the best interference effect and significant interference effect [35].

### qPCR detection of KDM1A expression levels in OCI-LY1 cells and cell model for DLD interference

qPCR was used to detect the changes in the expression level of KDM1A in the above two groups of cells (GAPDH as an internal reference) [36]. To ensure the reproducibility of the results, we set three replicates for each group.

### Cell cycle experiment

After corresponding culture stimulation, OCI-LY1 cells and culture medium will be transferred to centrifuge tubes. The centrifuge tubes will be centrifuged at 4 ℃ for 5 min (1000 rpm) and the supernatant will be removed, 3 ml of pre-cooled PBS was added to a centrifuge tube to resuspend cells. The centrifuge tube was centrifuged at 4 ℃ for 5 min (1000 rpm) and the supernatant was removed, pre-cooled 75% alcohol was added to a centrifuge tube to resuspend and fix the cells, and placed in a refrigerator at 4 ℃ for overnight fixation. Subsequently, pre-cooled PBS was added to the centrifuge tube and washed three times. The centrifuge tube was centrifuged at 4 ℃ for 5 min (1000 rpm) before removing the supernatant. Finally, Propidine iodide (PI) staining solution was added and stained in the dark at 37 ℃ for 30 min before flow cytometry detection.

 [37].

### Cell apoptosis experiment

Cells were collected (1 × 106 cells/time) and washed with pre-cooled PBS. Then we resuspended the cells using 1 ml 1X Binding Buffer and achieved a density of 1 × 106 cells/ml in the tube. Then we added 5 to the tube µ L Annexin V-FITC and gently mix for 10 min at room temperature and in dark conditions. Finally, we add 5 to the tube µ L PI was incubated at room temperature and in dark conditions for 5 min, and then detected by flow cytometry within 1 h [38].

### Statistical analysis

Differential gene expression’s most statistical analyses were performed using on-line databases, as mentioned above and R 4.2.1. Student t-test was used for comparison between the two groups. Kaplan Meier method was used for log rank test and survival analysis. For all results, *p* < 0.05 was considered statistically to be significant [39].

### Result

#### GEO database and machine learning used to screen key CRGs of COVID-19

**Fig. 1:**
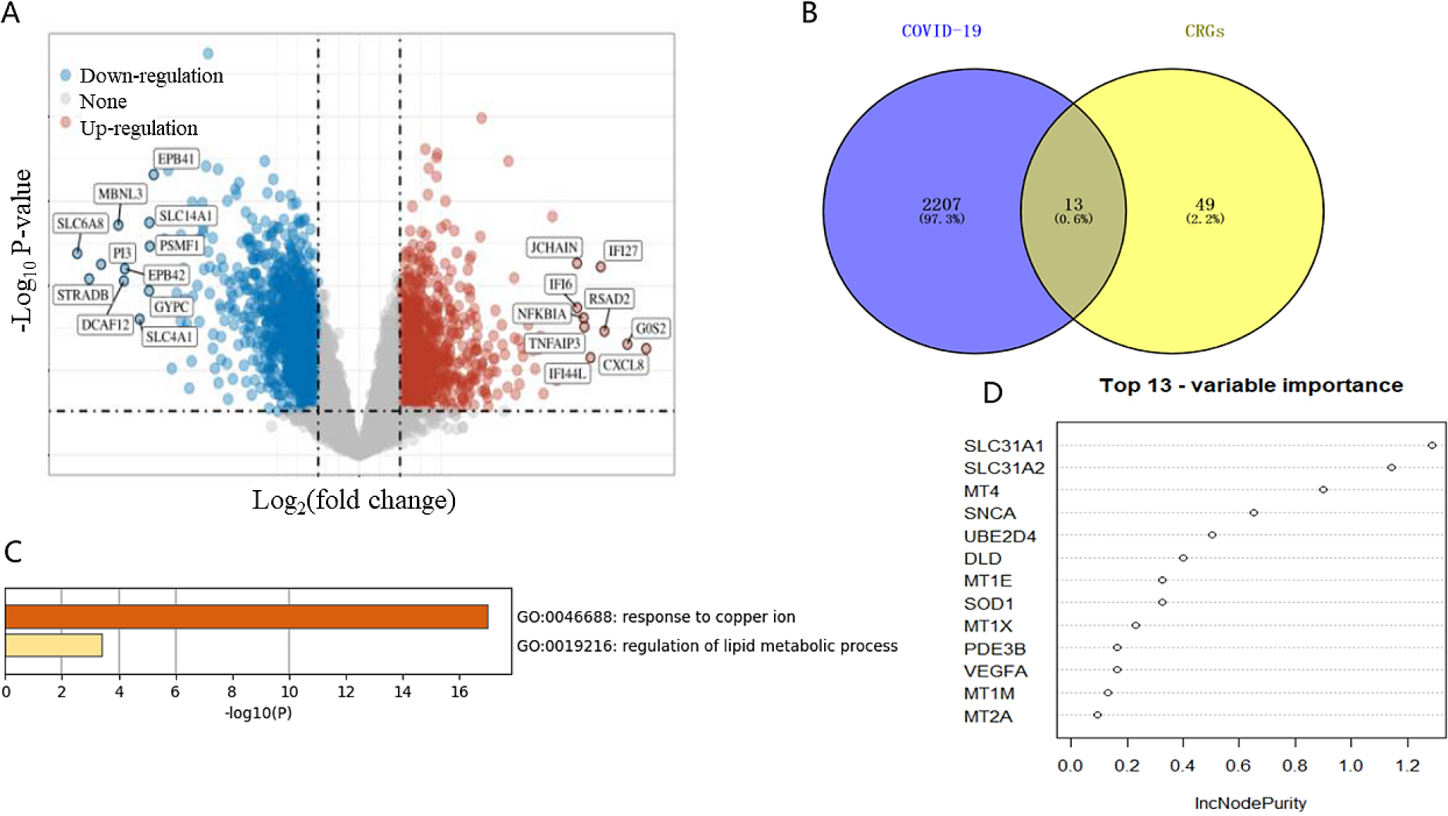
GSE177477 and machine learning used to screen key CRGs of COVID-19. The results were shown in a volcano plot, with red representing significantly upregulated differentially expressed genes and blue representing significantly downregulated differentially expressed genes (Fold change (FC) > 1.5 or FC < 0.67 and *P* < 0.05) (**A**). 13 Cuprotosis related genes (CRGs) were considered to have significant differences (**B**). 13 CRGs are mainly enriched in response to copper ions (*P* < 0.01) (C). Random Forest models were used to further screen the genes with the highest contribution to classification (IncNodePurity > 0.4) as key genes(D)

We obtained data from GEO177477 and conducted gene expression analysis. After data standardization, 13 CRGs were considered to have significant differences (Fig. 1A-B). 13 CRGs are mainly enriched in response to copper ions (Fig. 1C). Subsequently, RF models were used to further screen the genes with the highest contribution to classification (IncNodePurity > 0.4) as key genes. SLC31A1, SLC31A2, MT4, SNCA, UBE2D4, and DLD were considered to be the genes with the highest contribution to classification (Fig. 1D). Therefore, these six genes may be potential biomarkers for COVID-19.

### DLD is a potential biomarker for DLBCL tissue、peripheral blood and scRNA-seq

**Fig. 2:**
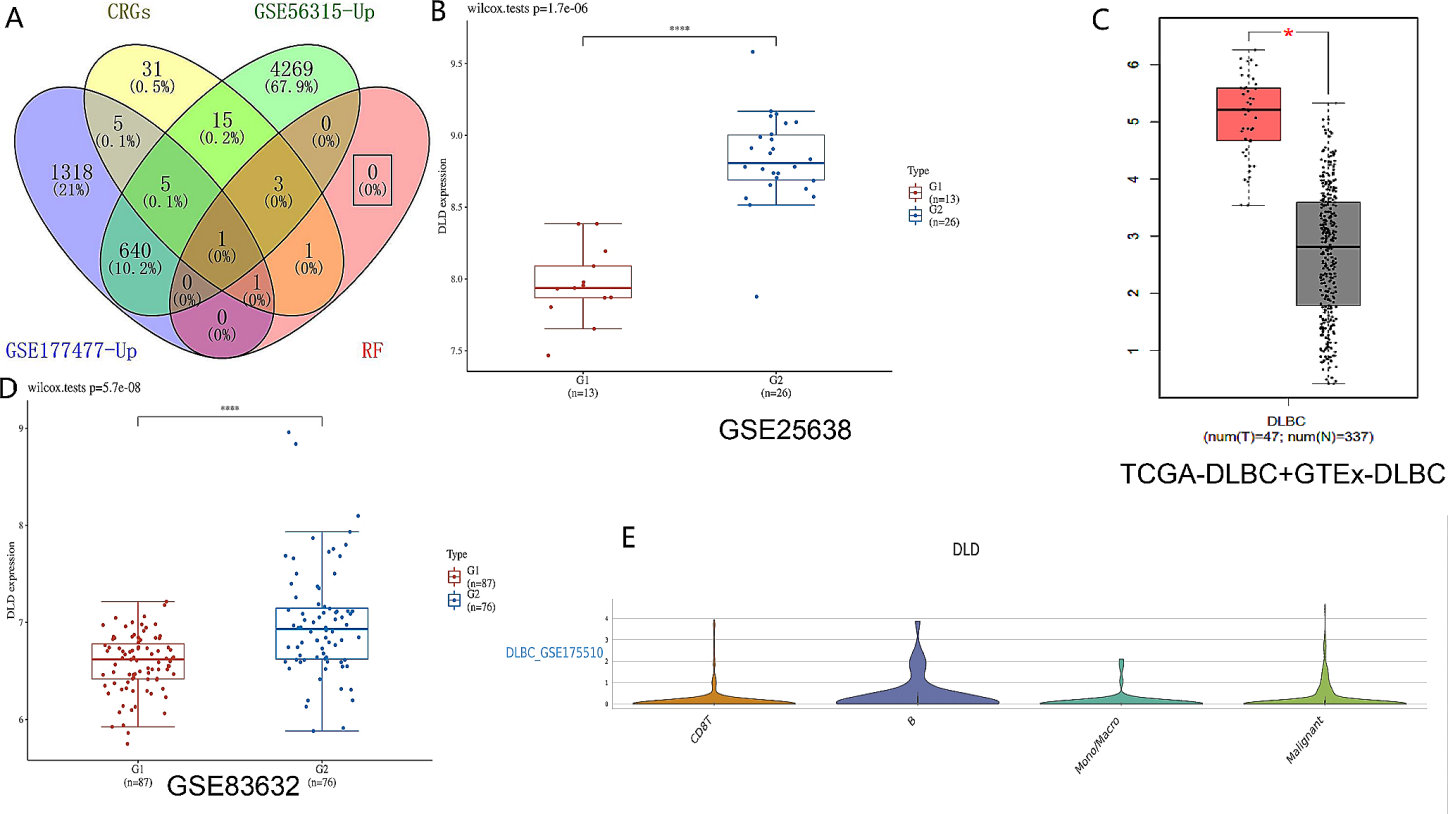
DLD is a potential biomarker for DLBCL tissue、peripheral blood and scRNA-seq. Dihydrolipoamide dehydrogenase (DLD) is a key up-regulated gene with significant differences between the two diseases(**A**). we validated the gene expression level of DLD in Diffuse large B-cell lymphoma (DLBCL) using GSE25638 (blue represents DLBCL, red represents control group samples) (**B**) and the online database Gene Expression Profiling Interactive Analysis (GEPIA) (red represents DLBCL, black represents control group samples) (**C**). DLD was significantly upregulated in peripheral blood samples of DLBCL (**D**). Based on the Tumor Immune Single-cell Hub(TISCH) database, single-cell sequencing was performed on an independent dataset of DLBCL to explore the correlation between immune cell distribution and DLD expression levels at the single-cell level. High levels of DLD expression were found in all four cells, particularly in CD8T cells, B cells, monocytes/Macrophages, and malignant cells (*E*)

We first used the COVID-19 sample from the GSE177477 dataset and the DLBCL sample from the GSE56315 dataset. Through univariate analysis and machine learning, we found that DLD is a key up-regulated gene with significant differences between the two diseases (Fig. 2A). Secondly, we validated the gene expression level of DLD in DLBCL using GSE25638 and the online database Gene Expression Profiling Interactive Analysis (GEPIA) (http://gepia.cancer-pku.cn/). According to this result, DLD is upregulated in cancer tissue compared to normal DLBCL tissue (Fig. 2B-C). Further analysis was conducted on the GSE83632 dataset of peripheral blood samples. We found that DLD was significantly upregulated in peripheral blood samples of DLBCL (Fig. 2D). Based on the Tumor Immune Single-cell Hub(TISCH) database(http://tisch.comp-genomics.org/), single-cell sequencing was performed on an independent dataset of DLBCL to explore the correlation between DLD expression levels and immune cell distribution at the single-cell level. High levels of DLD expression were found in all four cells, particularly in CD8T cells, B cells, monocytes/Macrophages, and malignant cells (Fig. 2E).

### The mutation landscape of DLD in DLBCL

Using the cBioPortal database to study DLD mutations in DLBCL. The results showed that the mutation frequency of DLD in DLBCL was 13% (Figure S2), indicating that DLD plays an important role in DLBCL. DLD mutations are significantly correlated with tumor mutation burden (TMB) (nonsynonymous), Race Category, Mutation Count, microsatellite instability (MSI) MANTIS Score and Sex (Table S1). Therefore, DLD mutations are associated with genomic heterogeneity in DLBCL.

### Pathway enrichment analysis at the transcriptional level of DLD and mechanism analysis at the protein level

**Fig. 3:**
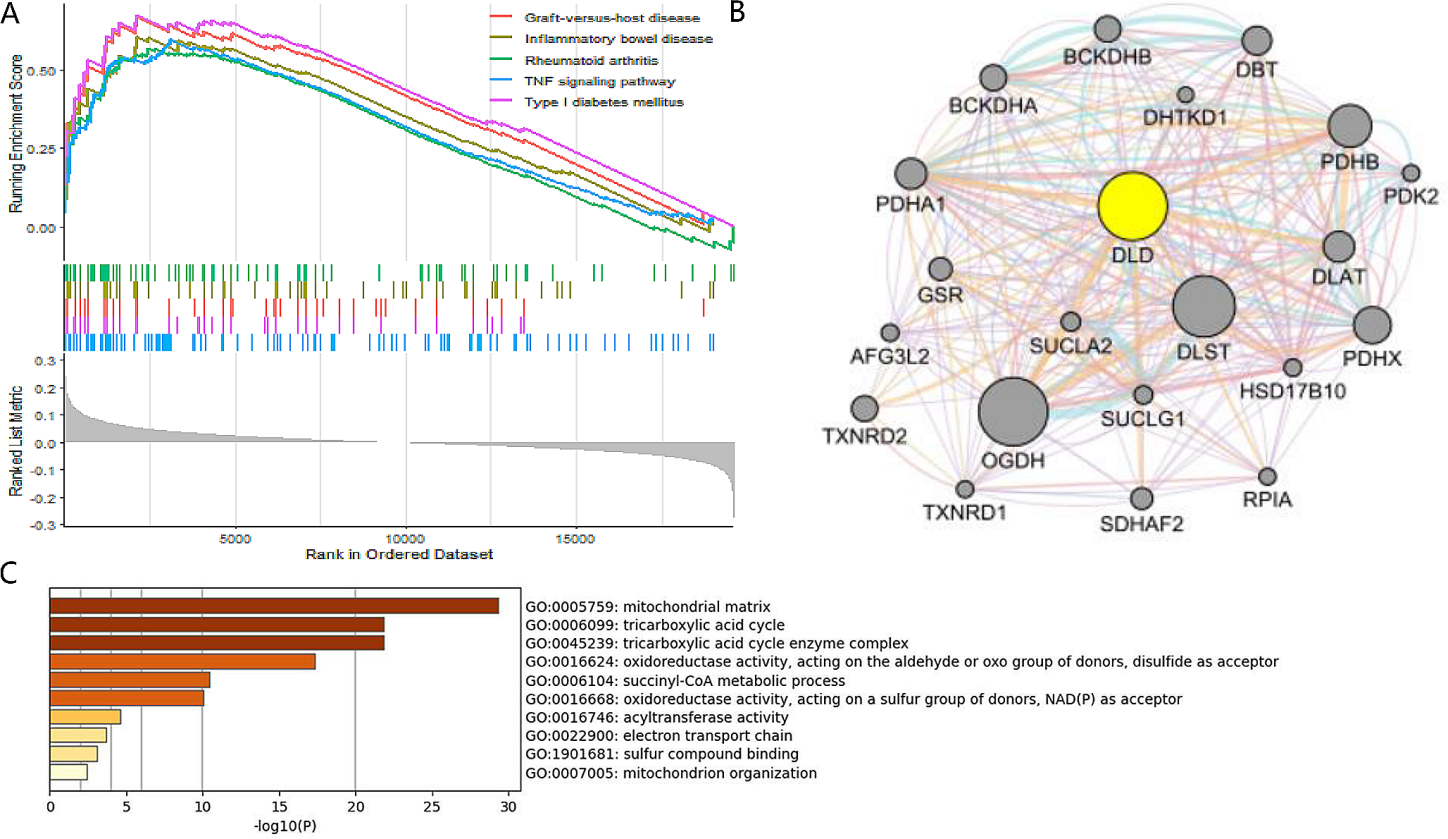
Pathway enrichment analysis at the transcriptional level of DLD and mechanism analysis at the protein level. The Gene Set Enrichment Analysis (GSEA) results showed that high-DLD was mainly enriched in Graft-versus-host disease, Inflammatory bowel disease, Rheumatoid arthritis, TNF signaling pathway (**A**). GeneMANIA was used to build a PPI network of 21 genes centered on DLD (**B**). Gene ontology (GO) function enrichment analysis were carried out for these 21 genes. Significantly rich GO terms include mitochondrial matrix, tricarboxylic acid cycle, tricarboxylic acid cycle enzyme complex (**C**)

The GSEA results showed that high-DLD was mainly enriched in Graft-versus-host disease, Rheumatoid arthritis, Inflammatory bowel disease, TNF signaling pathway (Fig. 3A). GeneMANIA was used to build a PPI network of 21 genes that were centered on DLD (Fig. 3B). GO function enrichment analysis were carried out for these 21 genes out. Significantly rich GO terms include tricarboxylic acid cycle, mitochondrial matrix, tricarboxylic acid cycle enzyme complex (Fig. 3C). These results suggest that DLD may be involved in DLBCL’s immune inflammation through the energy pathway and inflammatory signaling pathway.

### Correlation analysis between DLD and immune cell infiltration, immune checkpoints and immune characteristic genes

**Fig. 4:**
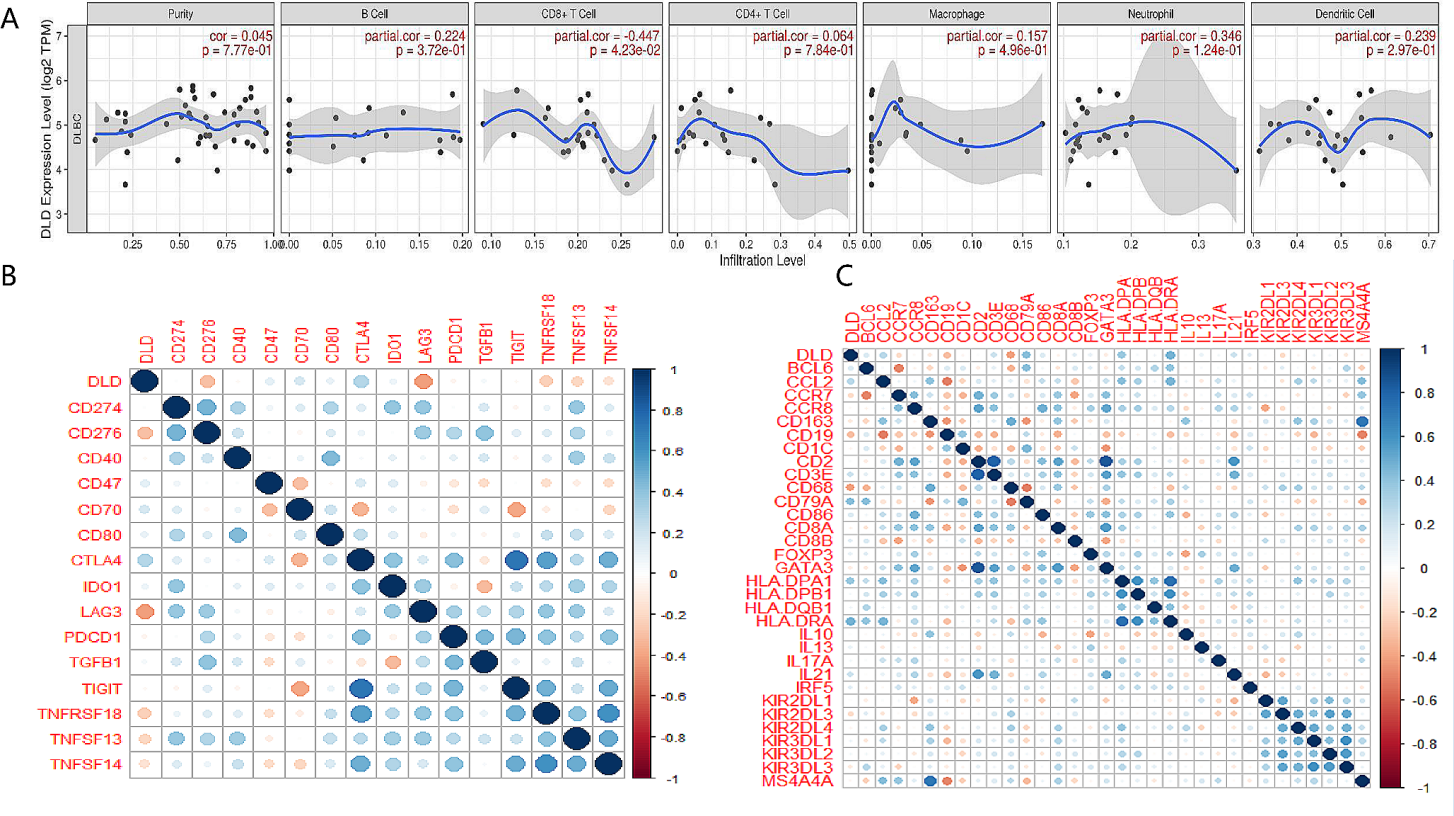
Correlation analysis between DLD and immune cell infiltration, immune checkpoints and immune characteristic genes. DLD is significantly negatively related to immune T Cells CD8 infiltration (**A**). The Spearman correlation analysis results showed that DLD was significantly positively correlated with immune checkpoint CD276 and LAG3 (*P* < 0.01) (**B**) and significantly correlated with B cell immune characteristic genes, TAM immune characteristic genes, and Dendritic cell immune characteristic genes(*P* < 0.05) (**C**)

Tumor microenvironment has been proved to play an important role in tumorigenesis. We used TIMER to determine whether the expression of DLD in DLBCL is related to immune cell infiltration. We found that DLD is significantly negatively related to immune T Cells CD8 infiltration (Fig. 4A). The Spearman correlation analysis results showed that DLD was correlated with LAG3 and immune checkpoint CD276 significantly positively (*P* < 0.01) (Fig. 4B) and correlated with B cell immune significantly characteristic genes, TAM immune characteristic genes, and Dendritic cell immune characteristic genes (*P* < 0.05) (Fig. 4C). LAG3 has been shown to be expressed in activated CD4 + cells and CD8 + T cells [40].

### Functional analysis of KDM1A in DLBCL and correlation analysis between DLD and KDM1A

**Fig. 5:**
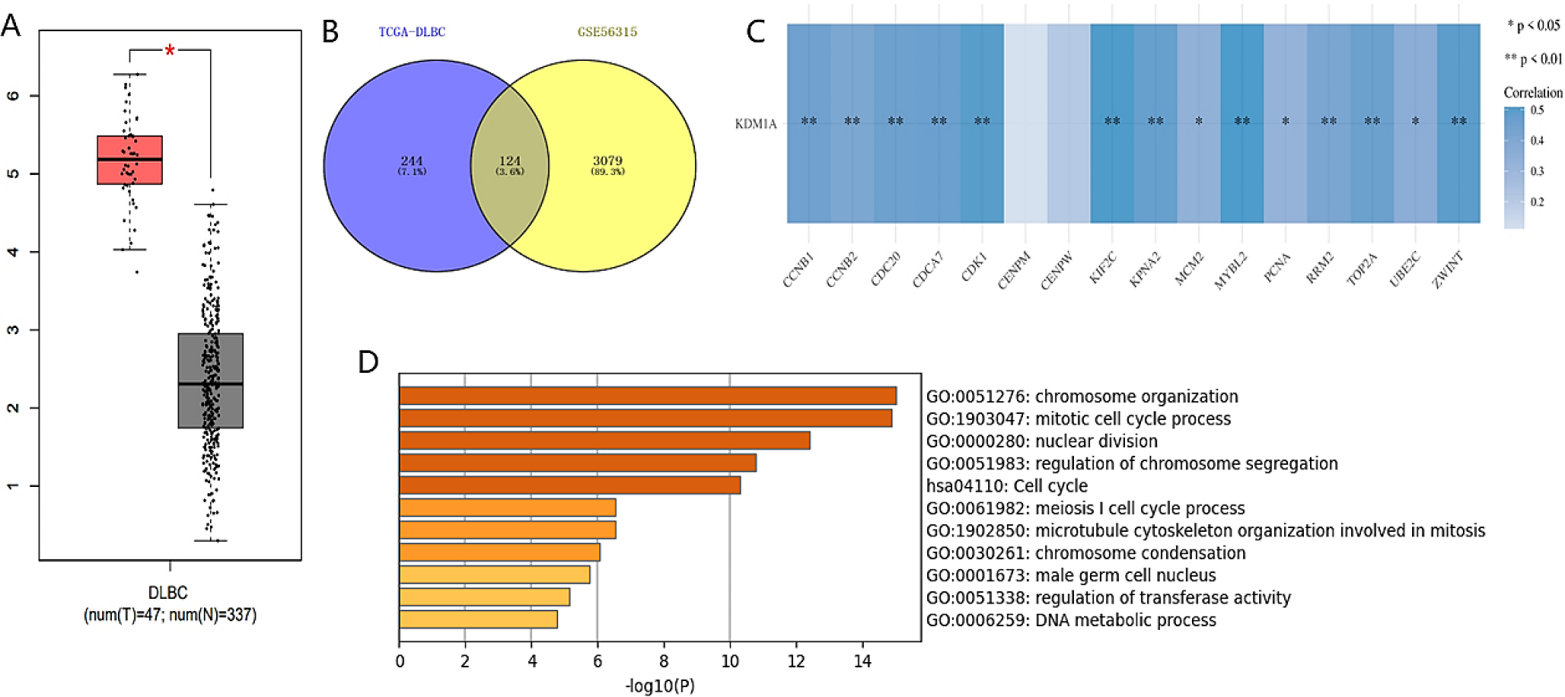
Functional analysis of KDM1A in DLBCL and correlation analysis between DLD and KDM1A. Compared with the control group sample, Lysine-specific demethylase 1 (LSD1, also known as KDM1A) were significantly expressed in DLBCL. Red represents the disease group, black represents the health group (*P < 0.01 and FC > 2) (**A**). By GSE56315, we analyzed the differential genes (*P* < 0.01 and Log2 > 4) that were up-regulated in DLBCL that ranked by The Cancer Genome Atlas (TCGA) data, and then verified them, A total of 124 differential genes have been verified (**B**). A total of 16 key MCODE genes were obtained and spearman correlation analysis was used to analyze the correlation between KDM1A and the 16 key MCODE genes of DLBCL, the results showed that 14 genes are correlated with KDM1A positively(Blue represents positive correlation) (**C**). The enrichment analysis results show that 14 genes are mainly enriched in the cell cycle pathway (**D**)

Compared with the control group sample, KDM1A were significantly expressed in DLBCL (Fig. 5A). Red represents the disease group, black represents the health group (FC > 2 and *P < 0.01). By GSE56315, we analyzed the differential genes (*P* < 0.01 and Log2 > 4) that were up-regulated in DLBCL that ranked by TCGA data, and then verified them, A total of 124 differential genes have been verified (Fig. 5B), and the key functional gene modules were obtained through the MCODE add-in in the Cytoscape software. We took the module with the highest total score (Score > 14), and a total of 16 key MCODE genes were obtained and spearman correlation analysis was used to analyze the correlation between KDM1A and the 16 key MCODE genes of DLBCL, the results showed that 14 genes are correlated with KDM1A positively (Fig. 5C). The enrichment analysis results show that 14 genes are mainly enriched in the cell cycle pathway, indicating that KDM1A regulates DLBCL cells’ cell cycle mainly (Fig. 5D). Finally, Spearman correlation analysis showed a significant positive correlation between DLD expression and KDM1A expression (Figure S2).

### Construction of cell model for DLD interference, qPCR detection of KDM1A expression levels in OCI-LY1 cells and cell model for DLD interference

**Fig. 6:**
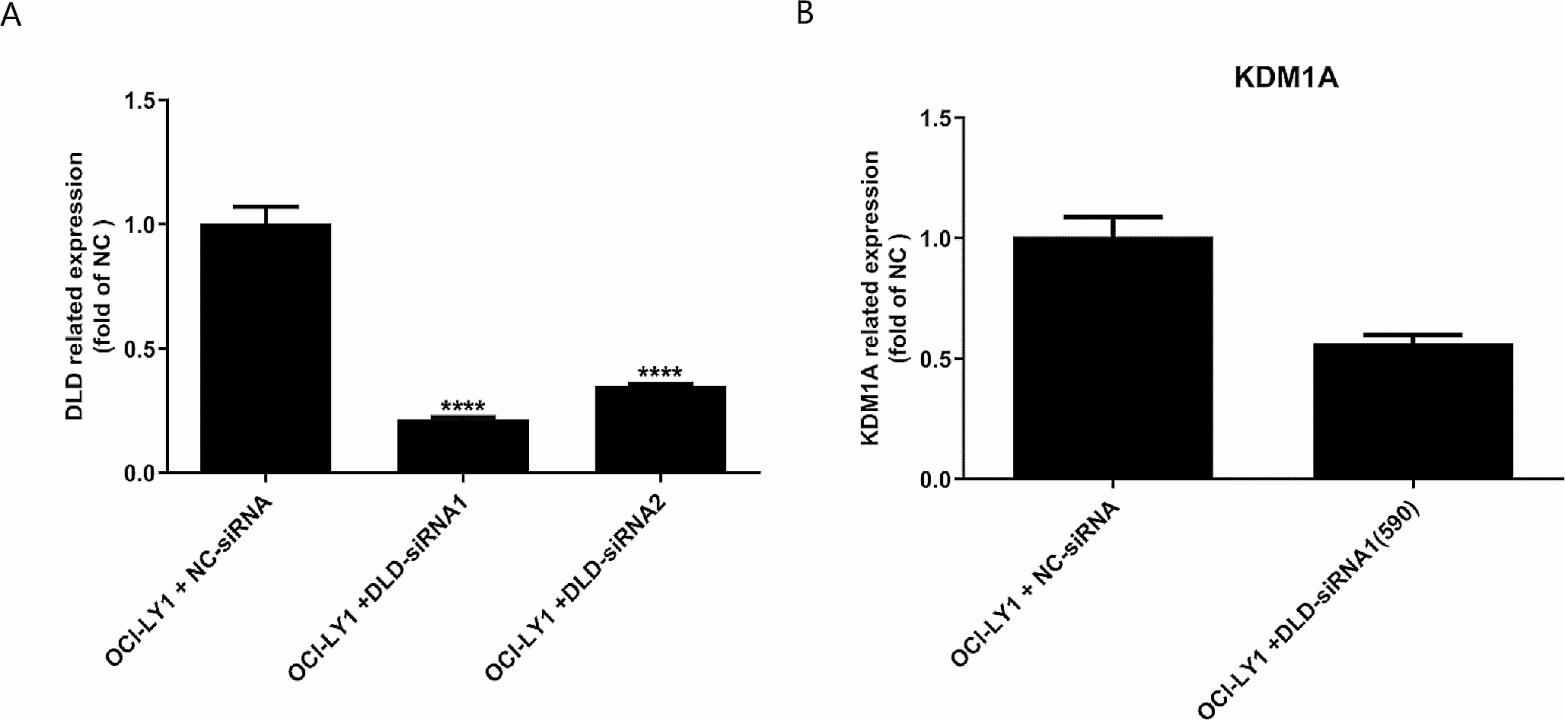
Construction of Cell Model for DLD Interference, qPCR detection of KDM1A expression levels in OCI-LY1 cells and Cell Model for DLD Interference. The qPCR results showed that compared with the control group, the cells transfected with OCI-LY1 + DLD-siRNA1 had the most significant interference effect on DLD (**A**). The qPCR results showed that compared with the control group, The KDM1A gene shows a significant downward trend in OCl-LY1 + DLD siRNA1 (590) (FC > 1.5) (**B**)

The qPCR results showed that compared with the control group, the cells transfected with OCI-LY1 + DLD-siRNA1 had the most significant interference effect on DLD (Fig. 6A). Therefore, the cells transfected with OCI-LY1 + DLD-siRNA1 were selected as the cell model for DLD interference and named OCI-LY1 + DLD-siRNA1(590). The qPCR results showed that compared with the control group, The KDM1A gene shows a significant downward trend in OCl-LY1 + DLD siRNA1 (590) (FC > 1.5) (Fig. 6B).

### Cell cycle experiment

**Fig. 7:**
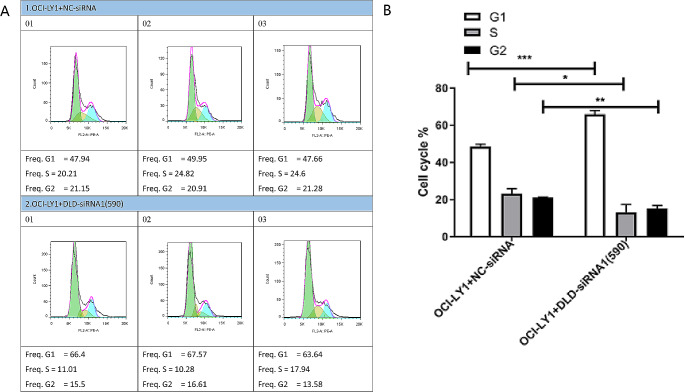
DLBCL Cell Cycle Experiment. The results of cell proliferation showed that compared with the OCI-LY1 + NC-siRNA group cells, the OCI-LY1 + DLD-siRNA1(590) group showed a significant increase in G1 phase cells, a significant decrease in S phase cells, and a significant decrease in G2 phase cells (**A, B**)

The results of cell proliferation showed that compared with the OCI-LY1 + NC-siRNA group cells, the OCI-LY1 + DLD-siRNA1(590) group showed a significant increase in G1 phase cells, a significant decrease in S phase cells, and a significant decrease in G2 phase cells (Fig. 7A, B). Therefore, the low expression of DLD significantly inhibited the progression of DLBCL cell cycle.

### Cell apoptosis experiment

**Fig. 8:**
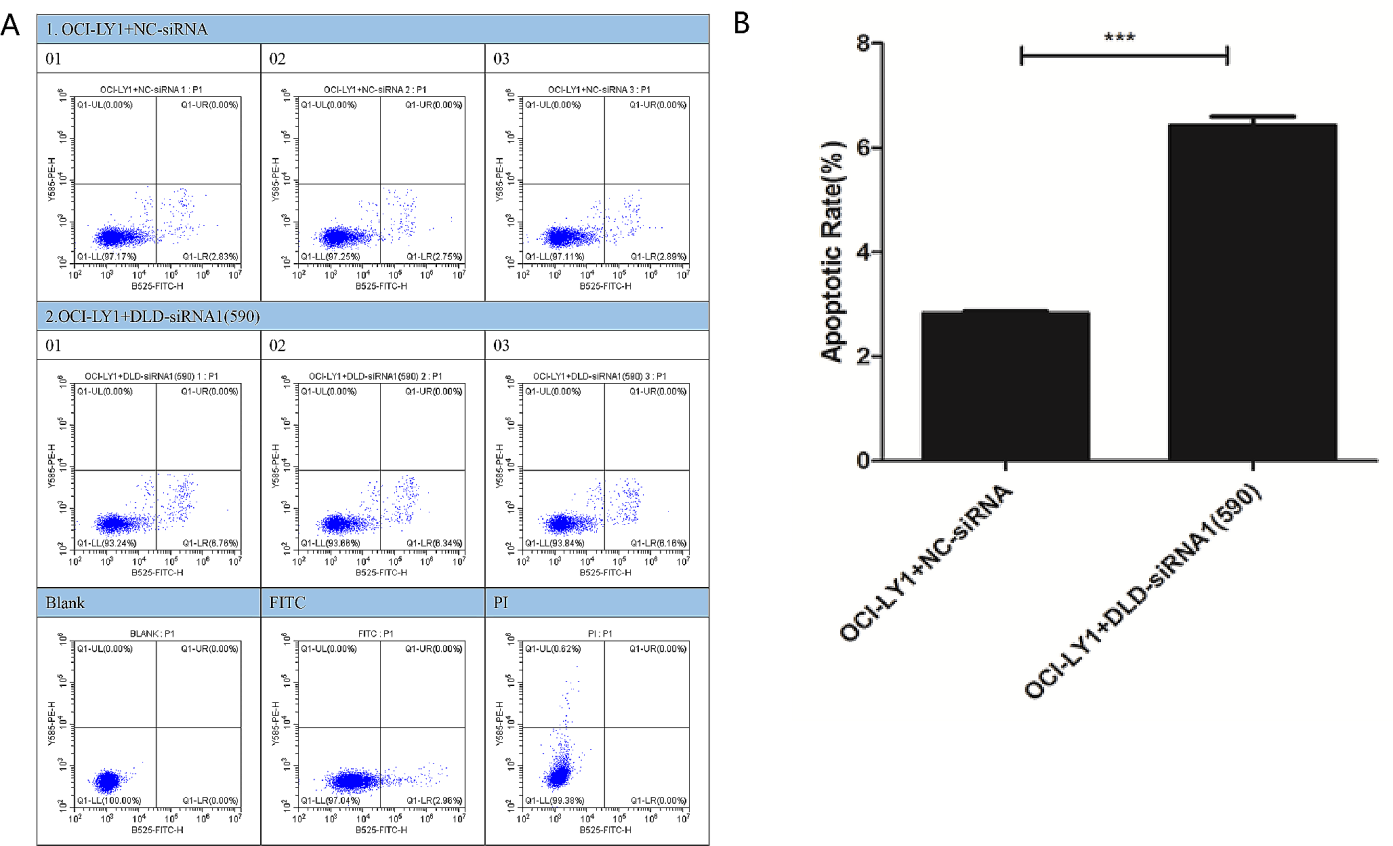
DLBCL cell apoptosis experiment. The results of cell apoptosis showed that compared with the OCI-LY1 + NC-siRNA group cells, the apoptosis rate of the OCI-LY1 + DLD-siRNA1(590) group cells was significantly increased (**A, B**)

The results of cell apoptosis showed that compared with the OCI-LY1 + NC-siRNA group cells, the apoptosis rate of the OCI-LY1 + DLD-siRNA1(590) group cells was significantly increased (Fig. 8A, B). Therefore, the low expression of DLD significantly promotes apoptosis of DLBCL cells.

#### Discussion

During the disease’s initial stages, COVID-19 patients had increased serum levels of copper ions, which were associated with the inflammatory response mainly [13]. It was found that whole blood copper levels were significantly higher in COVID-19 patients with severe condition compared to those with non-severe condition [14]. Research has found that serum copper in COVID-19 patients can help to predict patient prognosis, and supplementing copper adjuvants in patients that are diagnosed with copper deficiency may have a positive impact on disease outcomes [15]. Copper ion’s level therefore plays an important role in COVID-19. We found that the expression of 13 CRGs changed significantly and were significantly enriched in the copper ion pathway in COVID-19 (Fig. 1C). Therefore, 13 CRGs lead to copper homeostasis imbalance in COVID-19 based on the copper ion pathway. Copper ion levels’ imbalance can disrupt certain mitochondrial metabolic enzymes, which are more toxic in cells with active respiration. These enzymes can induce cell death, namely cuprotosis [16]. On the other hand, cuprotosis’ key signaling pathway, the FDX1 (Ferredoxin 1) -- LIAS (Lipic Acid Synthetase) axis, plays an important role in cellular oxidative stress’ regulation, oxidative stress can cause reactive oxygen species (ROS) production and cell apoptosis [10]. According to a report, in COVID-19, ROS rises [41], so 13 CRGs’ change causes copper ion level’s imbalance in COVID-19 patients, which induces cuprotosis, and further regulates oxidative stress through FDX1-LIAS axis, leading to ROS’s rise in the body. Our results are the first to identify 13 CRGs altering patients’ genetic characteristics potentially with COVID-19, and provided a theoretical basis for the treatment of COVID-19.

DLBCL has been reported as a risk factor for COVID-19, compared to healthcare providers with COVID-19, hospitalized DLBCL subjects with COVID-19 have more severe conditions and significantly higher risk of death [8], but the specific mechanism has not been elucidated. Our study found that six CRGs (SLC31A1, SLC31A2, MT4, SNCA, UBE2D4, DLD) play important roles in the pathological mechanism of COVID-19. DLD was significantly upregulated in both COVID-19 and DLBCL. Therefore, DLD may be a common pathological gene between COVID-19 and DLBCL.

DLD is a component of the glycine cleavage system and an E3 component of the three alpha-keto acid dehydrogenase complexes, DLD is mainly localized in the mitochondria and, to a lesser extent, in the nucleus [42]. DLD variants are involved in multiple diseases [43]. As a cuprotosis related gene, DLD has been confirmed by a lot of literature [44]. Cuprotosis through direct binding of copper with the lipoylation components of the TCA cycle, lipoylation is a key regulatory factor in cuprotosis, and DLD is a key gene encoding components of the lipoylation pathway, therefore, DLD regulates cuprotosis by encoding components of the lipoylation pathway (Figure S3) [10].We have demonstrated differential expression of DLD in DLBCL in organizational samples, peripheral blood samples, and single cells. Subsequently, we validated through cell experiments that DLD has a significant regulatory effect on cell cycle progression and apoptosis in DLBCL cells. Therefore, DLD can be a potential target for COVID-19 infection in DLBCL patients treatment.

KDM1A has been found to be highly expressed in colon cancer, breast cancer, neurocytoma and other tumor types, and high expression is associated with poor tumor prognosis [31]. Inhibiting KDM1A’s expression has been reported as a feasible tumor treatment strategy [32]. A study has found that KDM1A is overexpressed in human DLBCL tissue, KDM1A high-expression was significantly correlated with DLBCL size, International Prognostic Index and extra-nodal status [33]. Moreover, KDM1A expression was positive correlated with the expression of DLD (*R* = − 0.577, *P* = 0.004) (Figure S2). We detected KDM1A’s expression level through qPCR. The results significantly showed that low expression of DLD downregulated the KDM1A’s expression level in DLBCL-ABC type cells. Another study has confirmed that KDM1A knock-down in DLBCL-ABC type cells inhibited cell proliferation significantly and promoted cell apoptosis [45]. Our functional analysis results showed that 14 key genes are positively correlated with KDM1A. The enrichment analysis results show that 14 key genes are mainly enriched in the cell cycle pathway, indicating that KDM1A mainly regulates cell cycle of DLBCL cells. Compared with the OCI-LY1 + NC-siRNA group cells, the OCI-LY1 + DLD-siRNA1(590) group showed a significant rise in G1 phase cells, a significant decrease in S phase cells, and a significant decrease in G2 phase cells. DLD and KDM1A therefore have a crosstalk effect on DLBCL’s cell cycle pathway, and because KDM1A expression level is correlated with DLD expression level positively, and KDM1A has been proven to regulate the apoptosis and proliferation of DLBCL-ABC type cells. These data indicate that DLD regulates DLBCL cell proliferation and apoptosis by targeting and positively regulating KDM1A, providing potential therapeutic targets for DLBCL patients.

Current research shows that CD276 is a member of the negative B7 family, which inhibits T cell activation and plays a negative regulatory role in tumor immunity. CD276 can be expressed through nuclear factors- κ B (NF- κ B). The signaling pathways regulated by activating T cell nuclear factor (NFAT) and activating protein (AP) inhibit the proliferation of CD + 8 T cells and CD + 4 T cells, and reduce the levels of interleukin-2 (IL-2) and interferon- γ (IFN)- γ) the tumor’s secretion leads to tumor immune escape [46]. Research has found that LAG3 has a significant inhibitory effect on CD + 8 T cells activation [47]. Our research results show that DLD is correlated with CD276 and immune checkpoint LAG3 positively, and correlated with CD8 + T cells’ immune infiltration negatively.

DLD’s high expression may therefore reduce T cell-mediated anti-tumor immunity by regulating CD276 and immune checkpoints LAG3 positively and regulating CD8 + T cells’ immune infiltration. Reducing DLD’s expression can enhance T cell-mediated anti-tumor immunity effectively, thereby clearing cancer cells and preventing cancer growth.

There inevitably are several limitations to this study. This study comes from public databases mainly and is retrospective. The number of clinical information datasets that is available is limited, therefore the clinical parameters that are analyzed in this study are not comprehensive. DLBCL patients require real clinical information to determine DLD’s value. Secondly, although DLD’s function has been confirmed by DLBCL cells, its downstream and upstream mechanisms are not yet clear, and further experiments are needed to study its mechanism, which will be the research we need in the future.

### Conclusion

Since the discovery of cuprotosis in 2022, it has been one of the largest research hotspots. This study for the first time elucidated the molecular mechanism of CRGs in COVID-19 and screened out the six most important CRGs in COVID-19 through machine learning. Research has found that DLD is a differentially expressed gene shared by COVID-19 and DLBCL, therefore, DLD may be a key gene in promoting the occurrence and development of DLBCL by COVID-19. Down-regulation of DLD in DLBCL cells confirmed that DLD’s expression can inhibit DLBCL cells’ cycle progression significantly and promote cell apoptosis significantly. Further experiments showed that DLD regulates the apoptosis and proliferation of DLBCL cells by targeting and regulating KDM1A positively. This study for the first time elucidated CRGs’ molecular mechanism in COVID-19 and screened the six most important CRGs out in COVID-19 through machine learning. Research has found that DLD is a differentially expressed gene that is shared by DLBCL and COVID-19, DLD may therefore be a key gene in promoting the development and occurrence of DLBCL by COVID-19. Down-regulation of DLD in DLBCL cells confirmed that DLD’s expression can inhibit DLBCL cells’ cycle progression significantly and promote cell apoptosis significantly. Further experiments showed that DLD regulates the apoptosis and proliferation of DLBCL cells by targeting and regulating KDM1A positively. Immunoassay shows DLD’s high expression may reduce T cell-mediated anti-tumor immunity by regulating CD276 and immune checkpoints LAG3 positively and regulating CD8 + T cells’ immune infiltration. Reducing DLD’s expression can enhance T cell-mediated anti-tumor immunity effectively, thereby clearing cancer cells and preventing cancer growth. DLD may therefore be potential therapeutic target and a key gene for COVID-19 to promote DLBCL’s progression. Our research provides a theoretical basis for improving DLBCL’s clinical treatment.

## Electronic supplementary material

Below is the link to the electronic supplementary material.

Supplementary Material 1: Table S1: Mutation and clinical relevance of DLD in DLBCL

Supplementary Material 2

## Data Availability

No datasets were generated or analysed during the current study.

## Abbrevations

AP: Activating protein
COVID-19: Coronavirus disease 2019
Cuprotosis: Copper induces cell death
CRGs: Cuprotosis related genes
DLBC/DLBCL: Diffuse large B-cell lymphoma
DLD: Dihydrolipoamide dehydrogenase
FC: Fold change
FDX1: Ferredoxin 1
GEO: Gene Expression Comprehensive Database
GTEx: Genotype-Tissue Expression
GEPIA: Gene Expression Profiling Interactive Analysis
GO: Gene ontology
GSEA: Gene Set Enrichment Analysis
IL-2: Interleukin-2
IFN- γ: Interferon- γ
KEGG: Kyoto Encyclopedia of Genes
LSD1, also known as KDM1A: Lysine-specific demethylase 1
LIAS: Lipic Acid Synthetase
MSI: Microsatellite instability
NF- κ B: Nuclear factors- κ B
NFAT: Nuclear factor
PI: Propidine iodide
RF: Random Forest
TCA: Tricarboxylic acid
TCGA: The Cancer Genome Atlas
TISCH: Tumor Immune Single-cell Hub
TMB: Tumor mutation burden
