# Supplementary material for: DLD is a potential therapeutic target for COVID-19 infection in diffuse large B-cell lymphoma patients
Source: Apoptosis. 2024 Apr 6;29(9-10):1696–708. doi: 10.1007/s10495-024-01959-0 (PMC11416400; doi:10.1007/s10495-024-01959-0)
Supplement: Supplementary file 2 — Supplementary Material 2 [file 10495_2024_1959_MOESM2_ESM.docx]

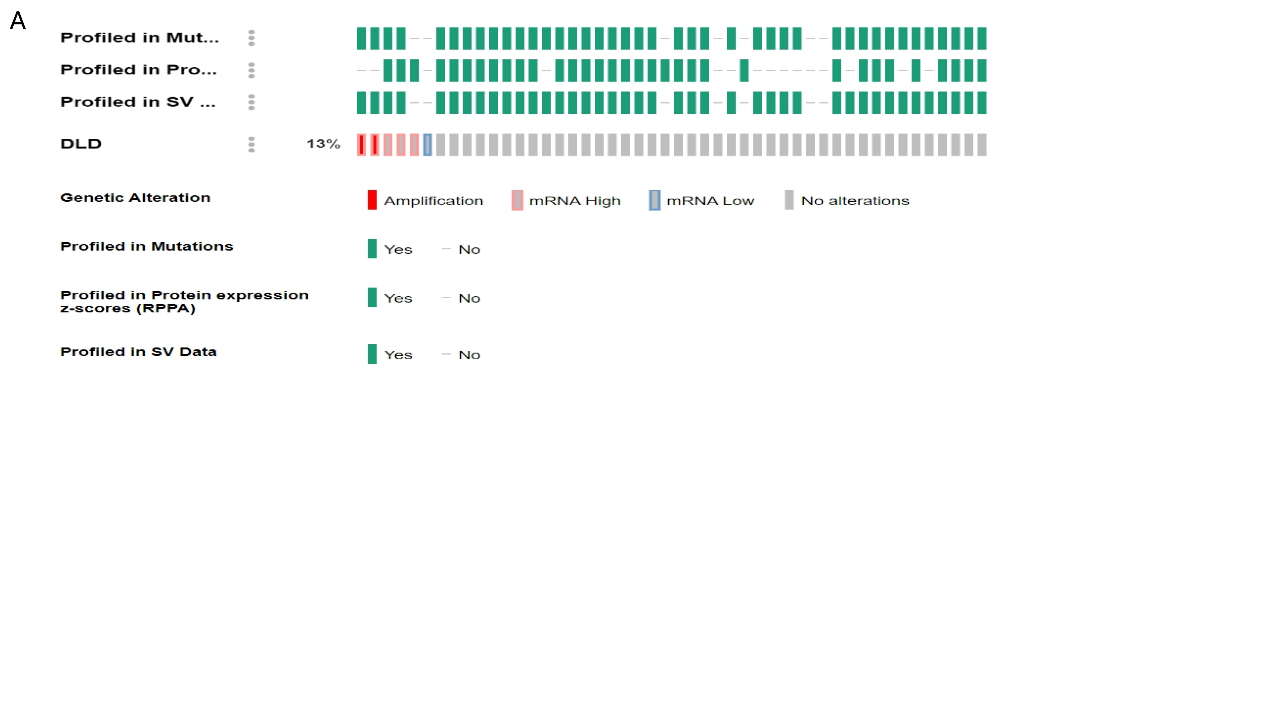


**Figure S1 The mutation landscape of DLD in DLBCL**

(A) The mutation frequency of DLD in DLBCL was 13%.


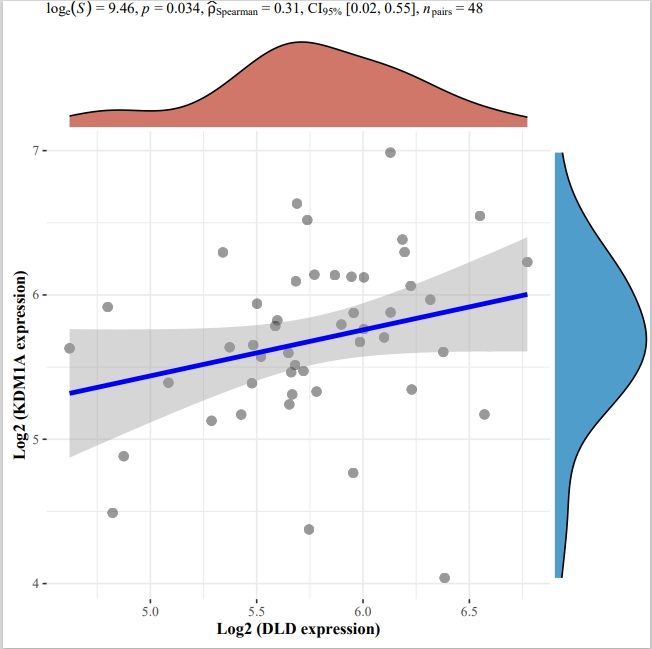


**Figure S2 Analyzing the mRNA expression level correlation between KDM1A and DLD based on the TCGA-DLBC dataset.**

Spearman correlation analysis showed a significant positive correlation between KDM1A and DLD at the mRNA expression level.


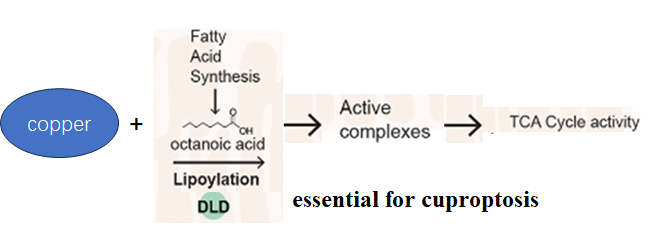


**Figure S3 DLD regulates cuprotosis pathway.**

DLD regulates cuprotosis by encoding components of the lipoic acid pathway, copper binds with lipoylated components to participate in the tricarboxylic acid (TCA) cycle, thereby inducing cuprotosis.
